# Managing What You Can’t Measure—Institutional Review Board Decision Support Software

**DOI:** 10.31486/toj.19.0074

**Published:** 2020

**Authors:** Stephen J. Rosenfeld

**Affiliations:** Freeport Research Systems, LLC, Freeport, ME

## INTRODUCTION

The well-worn adage “you can’t manage what you can’t measure” captures a particular view of management that has been reinforced by ideas such as continuous quality improvement. The idea that management means optimizing measurable outcomes makes intuitive sense and frees managers from the difficult task of managing people rather than processes. But this perspective is not easily applied to endeavors that are intrinsically difficult to quantitate and is even more difficult to apply without an explicit and widely accepted definition of quality*,* which in turn requires a common understanding of an organization's purpose.

In the world of institutional review boards (IRBs), the meaning and measurement of quality have been elusive targets. The purpose of the IRB is commonly seen as protecting research participants from harm, yet we do not have systems in place to capture the incidence of research-related harms. Even if we did, given the complexity of the research enterprise, we would be challenged to attribute such harms to the IRB. We could also argue that the purpose of the IRB is to ensure ethical research, which requires that possible benefits and harms are appropriately balanced in the context of the knowledge to be gained and that an individual's decision to participate is voluntary and informed. Thus, quantitation of research harms could not, and perhaps should not, provide a measure of the success of an IRB's mission.

Absent a common understanding of mission, IRBs are at risk of being perceived as little more than a compliance requirement burdening the research enterprise and, consistent with this view, IRB quality has been measured by metrics for the administrative process surrounding submission and review. Such processes are important, and the IRB community as a whole has benefitted from a focus on their improvement, but this focus leaves unaddressed the underlying question of quality and its relation to IRB decisions*.* In a world driven by metrics, there is even the possibility that the adage will be interpreted to suggest that if you can’t measure it, then it is not quality, implying that IRB decision-making is somehow arbitrary or not meaningful or, perhaps more accurately, that quality can be assumed given the proper setting. If we adopt this view, we should not be concerned with what goes on in IRB meetings as long as the board is appropriately constituted, receives its materials, and renders its determinations in a timely fashion.

Such an approach spares us the challenge of figuring out how to manage or improve what we cannot measure, but it does not reflect the experience of serving on an IRB. I suspect that IRB chairs and members have a sense that meetings and decisions can vary in quality, even if the quality cannot easily be assigned a number. Criticism of IRBs implicitly recognizes this aspect of quality in that IRBs are taken to task for the inconsistency of their decisions,^1^ an inconsistency that occurs both across boards and across time for a single board. While some variability is justifiable given the nature of the decisions an IRB is asked to make,^2^ it is fair to expect this variability to reflect differences in fact or ethical argument and not a decision-making process that is arbitrary.

These observations lead to the goal of managing and improving IRB decision-making. IRBs should be provided with tools to make decisions that are rationally justified by ethical principles and the facts of a particular review. Any decisions made in the absence of such facts will be arbitrary.

## SOFTWARE SUPPORT

To date, most software used to support IRBs has focused on the problem of collecting submission documents from sponsors and investigators. Stacks of protocols, consents, and investigator brochures have been replaced by workflow and document management systems that have greatly reduced the administrative burden on IRB members and staff and improved review process performance. Little concern or attention has been focused on what IRB members do with these documents beyond providing regulatory checklists to promote and document compliance. This lack of attention to tools that can improve decision-making is a missed opportunity to manage quality.

An analogy to electronic medical records (EMRs) provides a useful illustration of possible functionality. Like IRB software systems, early EMRs focused on solving the problem of availability of documents. Given the number of providers involved in caring for a patient in the hospital, the paper chart was often not available to a caregiver at the point of care. However, making every document relevant to a patient easily available had the perverse consequence of providing too much information, resulting in information overload and less time for reasoned clinical decisions. Only in later iterations did EMRs address the related problems of information overload and lack of decision support (eg, Cerner Corporation's promise that the “right data is presented in the right format at the right time”^3^). These later functions went beyond what could be achieved with the traditional paper medical record and were designed to improve the quality of clinical decision-making. Another useful parallel between EMRs and IRB systems is that EMRs are criticized for contributing to physician burnout, presumably because they are designed to address institutional administrative priorities (eg, to facilitate coding and charging) rather than to help physicians make better decisions and improve care. Interfaces are often cluttered with or dominated by data elements required for reimbursement and metrics tracking rather than care.^4^

IRB software is at the same stage as early EMR software. We have solved the problem of availability; many board members and staff can review the same material at the same time. But board members are often asked to complete data fields that are not directly related to ethical review to facilitate process improvement or to track data required by the institution, business, or accrediting body. Few features beyond document availability are provided to decrease distractions, focus deliberation on ethical determinations, and provide supporting or contextual information to enhance decision-making. Accordingly, three areas that should be addressed by IRB systems are context, history, and precedent.

## CONTEXT

No research stands by itself. By its nature, research builds on an existing foundation of knowledge that it aims to extend. From a regulatory perspective, 45 CFR §46.111(a)(2) of the Common Rule^5^ and the US Food and Drug Administration regulations^6^ require that “Risks to subjects are reasonable in relation to anticipated benefits, if any, to subjects and the importance of the knowledge that may reasonably be expected to result.”

An IRB must understand the extent of the “anticipated benefits” and “the importance of the knowledge that may … be expected to result.” A well-written protocol will provide the investigator's assessment of benefits and importance, but the IRB is empaneled to provide an independent assessment in the context of the involvement of human subjects, just as a review panel provides an independent assessment of the science in the context of the peer review process. This assessment should not be left to the investigator or sponsor, both of whom are likely to have understandable biases in favor of the research.

Before computers and the internet were available, such an independent assessment would have required a time-consuming literature review and/or a host of consultants on call. Computers allow access to clinical decision support tools such as Wolters Kluwer's UpToDate,^7^ as well as searches of literature databases, such as PubMed^8^ and Google Scholar.^9^ However, simply pointing out the availability of these resources to IRB members is not enough to make them useful. IRB review is a workflow, and tools will be used in proportion to their ease of use. Providing relatively simple classifications of research by intervention, targeted condition, and mechanism of action would allow IRB software to provide links to relevant material, and including the clinicaltrials.gov registration number would allow literature searches for reports related to the particular study.

**Figure 1.**
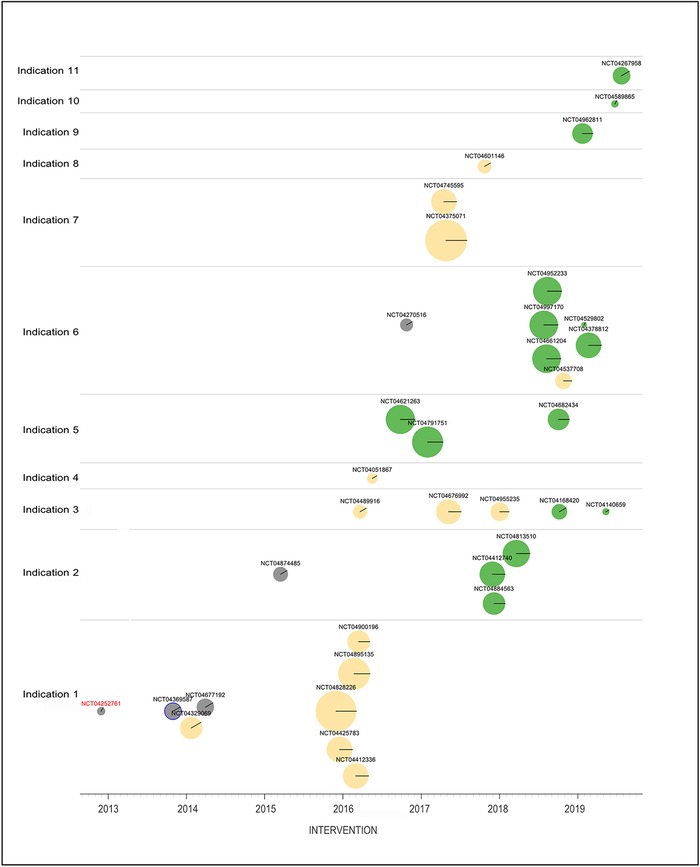
**In this context graphic, each circle represents a single study registered on clinicaltrials.gov of the intervention of interest. Studies are arranged horizontally by start date and vertically by the disorder being studied. The area of each study's disk is proportional to the planned or actual number of participants enrolled; the color of the disk indicates the status of the study (gray−completed, yellow−enrollment closed but study open, green−open and enrolling, red [not shown]−terminated). Studies with results reported on clinicaltrials.gov are enclosed in blue circles. Each disk also has a radial line corresponding to the clock-hand convention that indicates the phase of the study (eg, a phase 3 study has a radial at 3:00). Hovering the mouse over a disk reveals the full title of the study, and clicking a disk opens the corresponding webpage on clinicaltrials.gov. If a study has results, the results webpage will open.** Notes: This figure is adapted from review tools developed by the author for personal use in institutional review board review. The actual names of disorders and interventions and the clinicaltrials.gov NCT numbers have been replaced by placeholder text. A color version of this graphic is available at www.ochsnerjournal.org.

Clinicaltrials.gov^10^ is an important resource for understanding the context of a biomedical protocol. There is debate about how much responsibility the IRB has to avoid duplicative or unnecessary research,^11^ which is unethical because it exposes participants to unnecessary risks, but there is little debate about whether existing IRBs have the resources to discharge this responsibility—they do not. Capturing intervention and targeted condition information allows real-time discovery of prior and ongoing research, at least for research that meets the criteria for registration (Figure 1).

## HISTORY

Discussions of IRB quality typically focus on the 45 CFR §46.111 criteria for protocol approval,^5,12^ but the IRB will scrutinize a particular research project many times during its lifecycle. In addition to continuing review, required by 45 CFR §46.109(e)^5^ of the Common Rule, IRBs are also required by 45 CFR §46.108(4)(i) to review safety findings that might constitute “unanticipated problems involving risks to the subjects or others or any serious noncompliance….”.^5^ IRBs must also review amendments to the protocol and changes to the informed consent form and usually have the opportunity to review data and safety monitoring reports.

**Figure 2.**
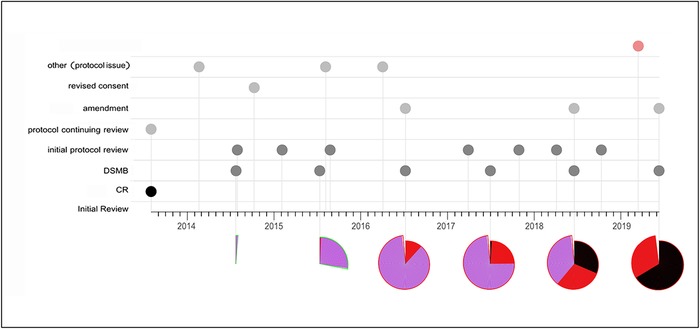
**The history graphic has two sections. In the top section, each filled disk on the timeline represents a protocol or review event. For review events, disks are color coded to indicate if the review took longer than expected. For example, a red disk is a signal that the review discussion was extended and the record of that review is likely to be informative in the review of the protocol as a whole. Disks are arranged horizontally by date of occurrence and vertically according to the nature of the event. Hovering the mouse over an event disk reveals the meeting notes for that event, and clicking a disk opens the full record of that review. The bottom section of the graphic depicts the enrollment status and history of the study at each scheduled continuing review. A fully enrolled study is shown as a complete circle. The border of the disk is green if enrollment was open at the time of continuing review and red if it was closed. The interior is divided into colored slices (purple−active participants, red−participants who were withdrawn, and black−participants who have completed the study). As the graphic shows, enrollment typically begins with active participants, and over time, the number withdrawn or completed dominates.** Notes: This figure is adapted from review tools developed by the author for personal use in institutional review board review. A color version of this graphic is available at www.ochsnerjournal.org.

Given the workload of many IRBs and the unpredictable timing of many of these events, the IRB members participating in a review may not be the same members who reviewed the protocol's initial submission. While the document management nature of IRB software theoretically allows board members to look at earlier submission materials and minutes, de novo review of the former is labor intensive and review of the latter is typically uninformative, as 45 CFR §46.§115(2)^5^ only requires minutes to contain the rationale for controverted decisions, and minutes often contain the minimal information to meet the regulations, presumably for liability reasons. Providing a bird's-eye view of prior reviews is a straightforward software function and can give board members a sense of the protocol's progress and access to prior rationales (Figure 2). One of the criticisms of IRBs is that they render inconsistent determinations,^2^ but such inconsistency is to be expected without easy access to prior decisions and their rationales.

## PRECEDENT

**Figure 3.**
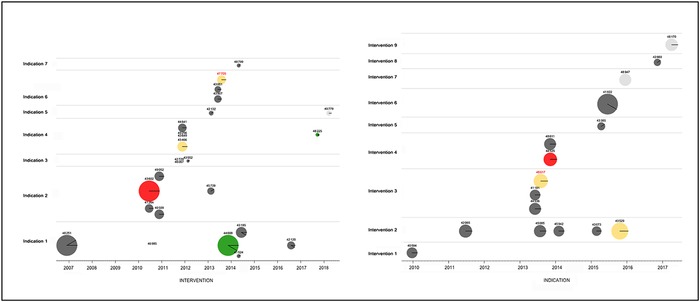
**The precedent graphic shows other reviewed studies involving the same intervention (left panel) and indication (right panel). In the intervention panel, studies are organized horizontally by date of initial review and vertically by indication. In the indication panel, studies are organized horizontally by date of initial review and vertically by intervention. Hovering the mouse over a study disk reveals the full name of the study, and clicking a study disk opens the full review record. Similar graphics or actionable (clickable) text lists can be generated for studies that are similar in level of risk, use of placebo, or inclusion of minors.** Notes: This figure is adapted from review tools developed by the author for personal use in institutional review board review. The actual interventions and indications in both panels have been replaced by placeholder text. A color version of this graphic is available at www.ochsnerjournal.org.

IRBs make reasoned ethical decisions about the factual circumstances of a particular research protocol at a particular time. Such decisions could serve as a record of the evolving ethical norms of society around new issues and technologies (eg, privacy, CRISPR, comparative effectiveness research, and cluster randomization). However, instead of building a record of ethical decisions, IRBs operate in isolation with little access to their own earlier decisions and no ability at all to learn from the reasoning of other IRBs. Suggesting that all IRBs contribute to a body of ethical precedent may be aspirational, but IRBs should be expected to operate in the full knowledge of decisions they have made in the past. An IRB's decisions on similar issues should be consistent over time or, if not consistent, the variability should be justified by the facts of the protocol or by new ethical arguments. Again, the capabilities of computers could provide easy access to past decisions. If relatively minor effort is expended to categorize protocols by criteria such as intervention, targeted condition, risk level, and phase, data visualization tools and databases could provide an IRB with ready access to comparators and visibility into prior ethical reasoning (Figure 3).

## CONCLUSION

Computers need to be seen as tools or cognitive extenders. They do not simply automate existing processes; they also provide capabilities to access a universe of resources to make better decisions. Computers also allow the presentation of data in ways that would be impossible or at least extremely laborious if done manually. It is time to apply these capabilities to the task of IRB decision-making. Doing so will result in higher quality, more consistent decisions that are backed by defensible, ethical reasoning.
